# Supplementary material for: PD-1/CD80+ small extracellular vesicles from immunocytes induce cold tumours featured with enhanced adaptive immunosuppression
Source: Nat Commun. 2024 May 8;15:3884. doi: 10.1038/s41467-024-48200-9 (PMC11079016; doi:10.1038/s41467-024-48200-9)
Supplement: Supplementary file 2 — Supplementary Information [file 41467_2024_48200_MOESM2_ESM.pdf]

Supplementary Information

PD-1/CD80$^+$ small extracellular vesicles from immunocytes induce cold tumours featured with enhanced adaptive immunosuppression

Lin-Zhou Zhang[1,#], Jie-Gang Yang[1,2,#], Gai-Li Chen[3], Qi-Hui Xie[1,2], Qiu-Yun Fu[1], Hou-Fu Xia[1,2], Yi-Cun Li[4], Jue Huang[1], Ye Li[1], Min Wu[1], Hai-Ming Liu[1], Fu-Bing Wang[5], Ke-Zhen Yi[6], Huan-Gang Jiang[3], Fu-Xiang Zhou[3], Wei Wang[7], Zi-Li Yu[1,2], Wei Zhang[1,2], Ya-Hua Zhong[3], Zhuan Bian[1], Hong-Yu Yang[4], Bing Liu[1,2], Gang Chen[1,2,8,9*]

[1]State Key Laboratory of Oral & Maxillofacial Reconstruction and Regeneration, Key Laboratory of Oral Biomedicine Ministry of Education, Hubei Key Laboratory of Stomatology, School & Hospital of Stomatology, Wuhan University, Wuhan 430079, China.

[2]Department of Oral and Maxillofacial Surgery, School and Hospital of Stomatology, Wuhan University, Wuhan 430079, China.

[3]Department of Radiation and Medical Oncology, Hubei Key Laboratory of Tumour Biological Behaviors, Hubei Cancer Clinical Study Center, Zhongnan Hospital of Wuhan University, Wuhan 430071, China.

[4]Department of Oral and Maxillofacial Surgery, Peking University Shenzhen Hospital, Shenzhen 518036, China.

[5]Department of Laboratory Medicine and Center for Single-Cell Omics and Tumour Liquid Biopsy, Zhongnan Hospital of Wuhan University, Wuhan 430071, China.

[6]Department of Laboratory Medicine, Zhongnan Hospital of Wuhan University, Wuhan 430071, China.

[7]Department of thoracic surgery, Renmin Hospital of Wuhan University, Wuhan 430060, China.

[8]TaiKang Center for Life and Medical Sciences, Wuhan University, Wuhan 430071, China.

[9]Frontier Science Center for Immunology and Metabolism, Wuhan University, Wuhan 430071, China.

[#]These authors contributed equally.

*Correspondence: geraldchan@whu.edu.cn (G.C.)

Supplementary figures

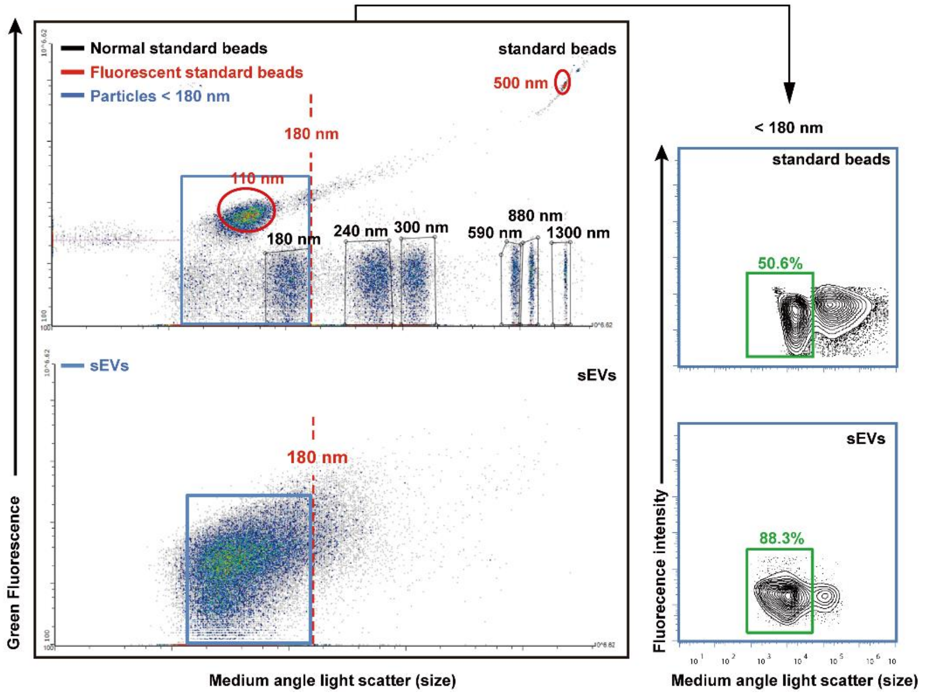

Supplementary Fig. 1 A micro flow cytometer with the indicated gate strategy

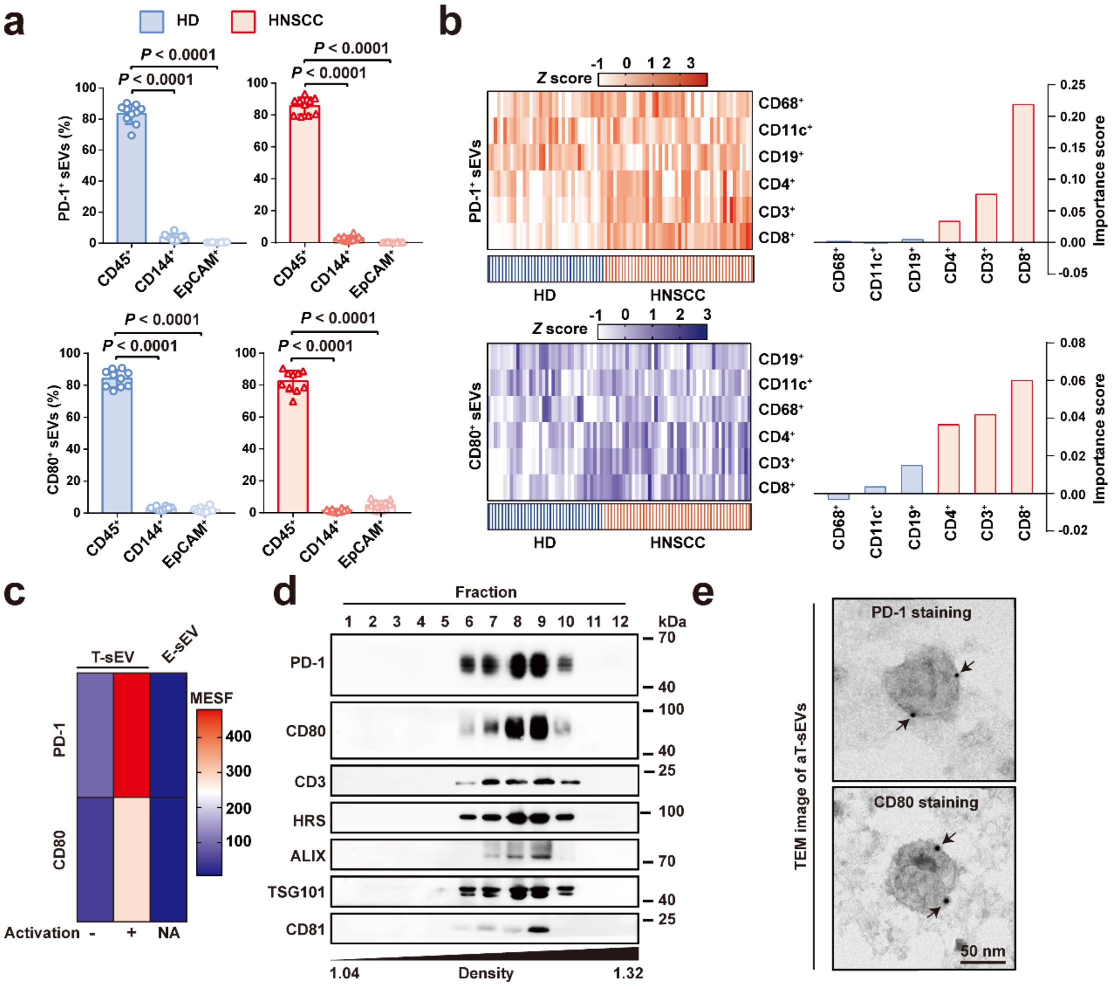

Supplementary Fig. 2 Circulating sEV PD-1/CD80 were mainly derived from activated immunocytes

a The proportions of the PD-1- or CD80-positive circulating sEVs co-stained with CD45, CD144 or EpCAM in HD (n = 10) and HNSCC patients (n = 10). **b** Heat maps illustrated the relative levels of immunocyte markers (CD3, CD4, CD8, CD11c, CD19, and CD68) on circulating PD-1$^+$ and CD80$^+$ sEVs from HD (n = 36) and HNSCC patients (n = 46) (left). Blue and red hatches indicate HD and HNSCC, respectively. Bar plots showed the importance score of immunocyte-derived sEV PD-1/CD80 in distinguishing HNSCC patients from HDs (right). HD, healthy donor. **c** Heat map of the relative levels of sEV PD-1 and C80 from T cells and tumour cells based on analysis of molecules of equivalent soluble fluorochrome (MESF). **d** Iodixanol density gradient centrifugation analysis showed that PD-1 and CD80 secreted by activated T

cells co-fractionated with sEV markers HRS, ALIX, TSG101 and CD81 and T cell-specific marker CD3. **e** Representative TEM image from activated T cell-derived sEVs immunogold-labelled with anti-PD-1 antibodies (top) and anti-CD80 antibodies (bottom). Arrowheads indicated 5 nm gold particles. Scale bar, 50 nm. For **a** Data were presented as mean ± s.d.; One-way ANOVA. The relevant raw data and uncropped blots are provided as a Source Data file.

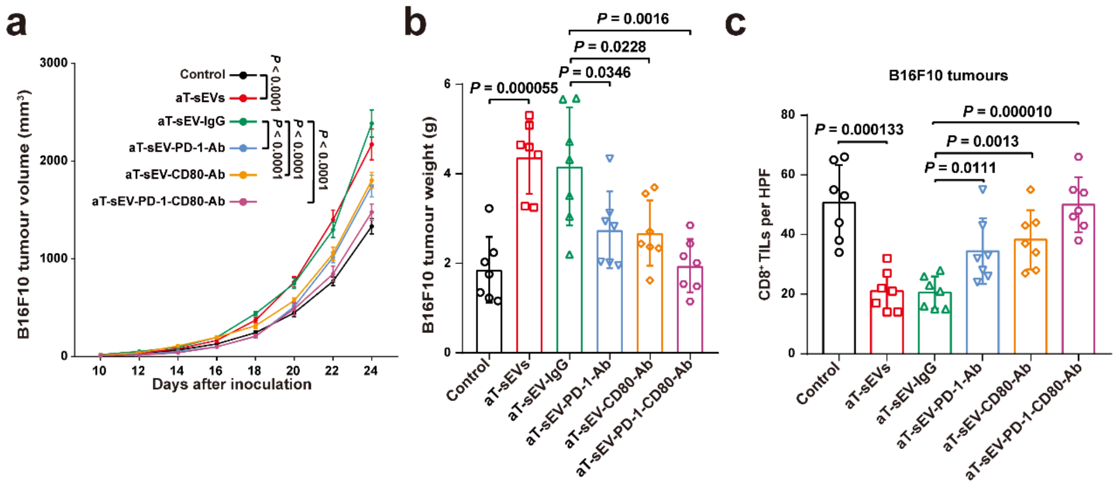

Supplementary Fig. 3 Circulating sEV PD-1/CD80 were associated with tumour growth in mice model

a Growth curve of B16F10 tumours in C57BL/6 mice after with indicated treatments (n = 7). **b** Weights of B16F10 tumours in C57BL/6 mice after in indicated treatments (n = 7 mice per group). **c** Quantification for the number of $CD8^{+}$ TILs in B16F10 tumours (n = 7 mice per group). For **a** data were presented as mean ± S.D.; Two-way ANOVA. For **b** and **c** data were presented as mean ± S.D.; Two-sided *t*-test. Source data are provided as a Source Data file.

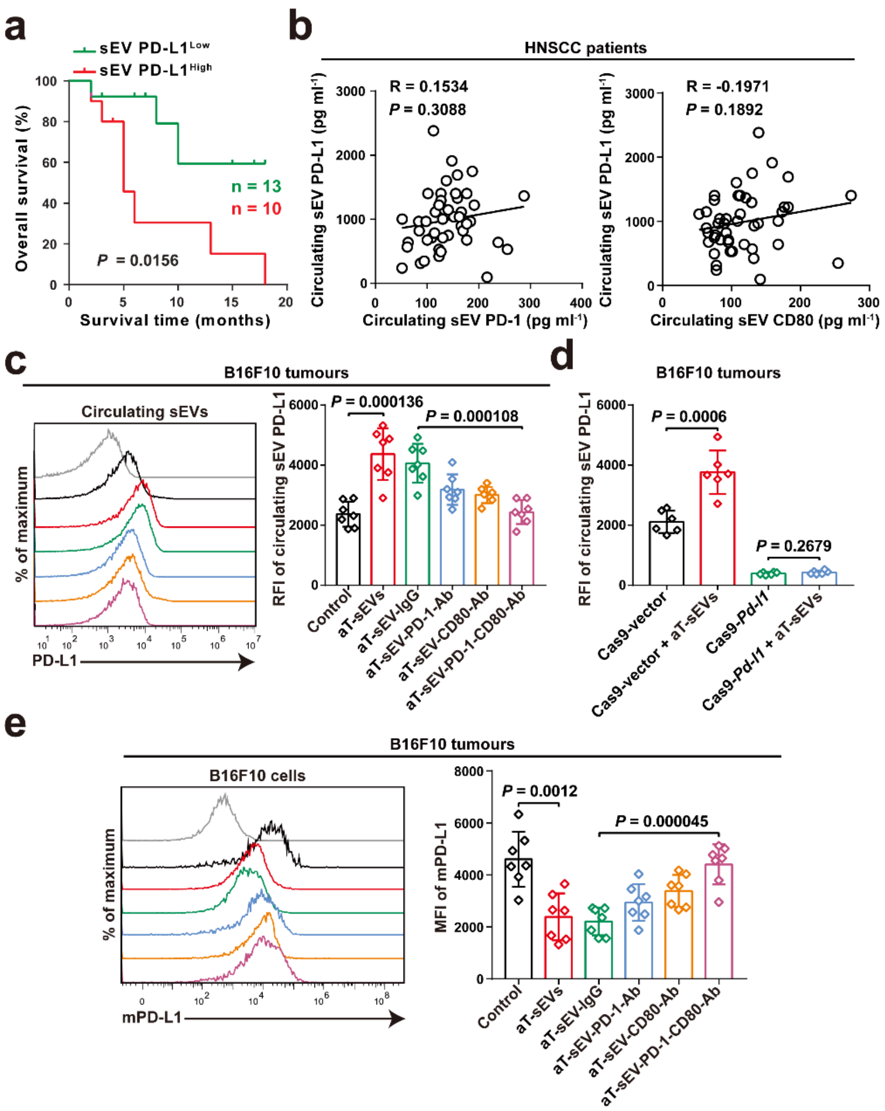

Supplementary Fig. 4 The levels of circulating sEV PD-1/CD80 were not correlated with that of sEV PD-L1 in patients with PD-L1-negative tumour cells

a Overall survival for patients with high (n = 10) and low (n = 13) levels of circulating sEV PD-L1. Log-rank test. **b** Pearson correlation of the levels of circulating sEV PD-L1 with circulating sEV PD-1 and CD80 in HNSCC patients (n=46). **c** Representative histograms (left) and quantification analysis (right) of PD-L1 labelled circulating sEVs from C57BL/6 mice bearing B16F10 tumours after with indicated treatments (n = 7 mice per group). **d** Quantification for the circulating sEV PD-L1 in plasma samples from C57BL/6 mice bearing B16F10-Cas9-vector and B16F10-Cas9-*Pd-l1* xenograft after in indicated treatments (n

= 6 mice per group). **e** Representative histograms (left) and quantification analysis (right) of proportion of mPD-L1 positive B16F10 cells after with indicated treatments (n = 7 mice per group). mPD-L1, membrane PD-L1. For **c**, **d**, and **e** data were presented as mean ± S.D.; Two-sided *t*-test. The relevant raw data are provided as a Source Data file.

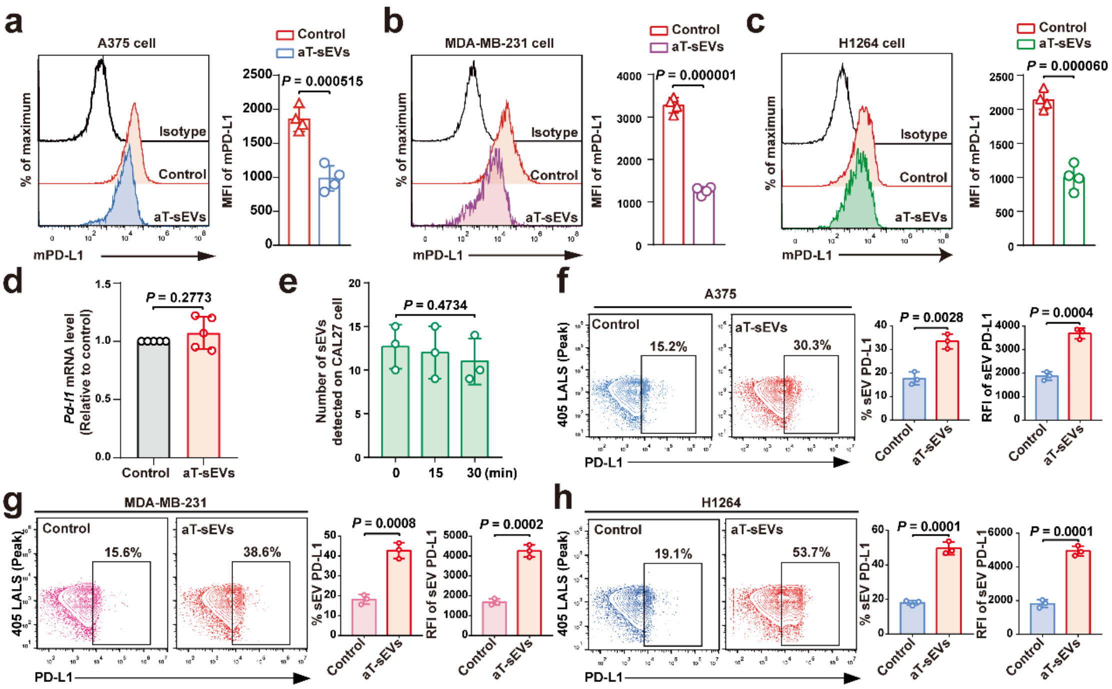

Supplementary Fig. 5 PD-1/CD80 on I-sEVs induced universal internalization of membrane PD-L1 and sEV PD-L1 secretion in tumour cells

a-c Representative flow cytometric histograms and quantification analysis of mPD-L1 expression level in tumour cells including A375 (**a**), MDA-MB-231 (**b**) and H1264 (**c**) with or without aT-sEV treatment. MFI, mean fluorescence intensity. **d** Quantification for the relative *PD-L1* mRNA level in CAL27 cells with or without T-sEV treatment. aT-sEVs, activated T cell-derived sEVs. **e** Quantification for the number of CFSE-labelled aT-sEVs bound to membrane of CAL27 cells. **f-h** Representative contour plots and quantification analysis of the level of sEV PD-L1 secreted from tumour cells including A375 (**f**), MDA-MB-231 (**g**) and H1264 (**h**) with or without aT-sEV treatment. For **a**, **b**, **c**, **d**, **e**, **f**, **g**, and **h** data were presented as mean ± S.D.; n ≥ 3 biologically independent samples; Two-sided *t*-test. Source data are provided as a Source Data file.

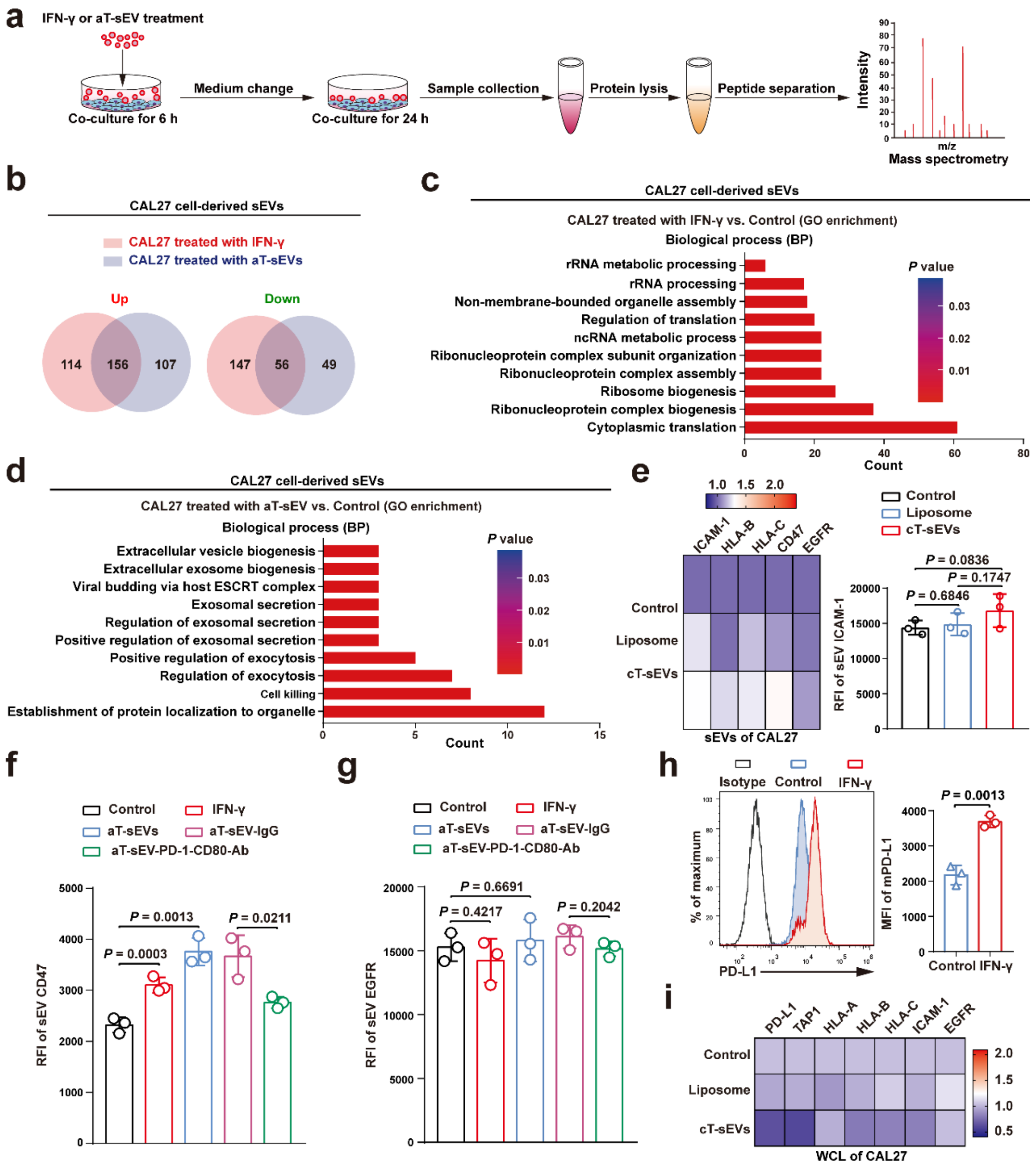

Supplementary Fig. 6 IFN-γ and I-sEVs regulated adaptive immune in different ways

a Schematic for mass spectrometry analysis of sEVs secreted by CAL27 cells with indicated treatment. **b** Venn diagrams showed the overlap proteins in sEVs secreted from CAL27 with IFN-γ or aT-sEV treatment. **c-d** GO enrichment analysis of DEGs in sEVs secreted from CAL27 after treatment of IFN-γ (**c**) or aT-sEVs (**d**). GO, gene ontology; DEGs, differentially expressed genes. **e** Heat map illustrated the relative levels of ICAM-1, HLA-B, HLA-C, CD47, and EGFR

in CAL27 cell-derived sEVs after with indicated treatments (left). Quantification analysis of the level of ICAM-1 in CAL27 cell-derived sEVs after treatment of liposomes or cT-sEVs (right). **f**-**g** Quantification analysis of the levels of CD47 (**f**) and EGFR (**g**) in CAL27 cell-derived sEVs after with indicated treatments. **h** Representative flow cytometric histograms (left) and quantification analysis (right) of the expression level of mPD-L1 in CAL27 cells treated with IFN-γ. **i** Heat map illustrated the relative level of PD-L1, TAP1, HLA-A, HLA-B, HLA-C, ICAM-1, and EGFR in CAL27 cells after in indicated treatments. For **e**, **f**, **g**, and **h** data were presented as mean ± S.D.; n = 3 biologically independent samples; Two-sided *t*-test. The relevant raw data are provided as a Source Data file.

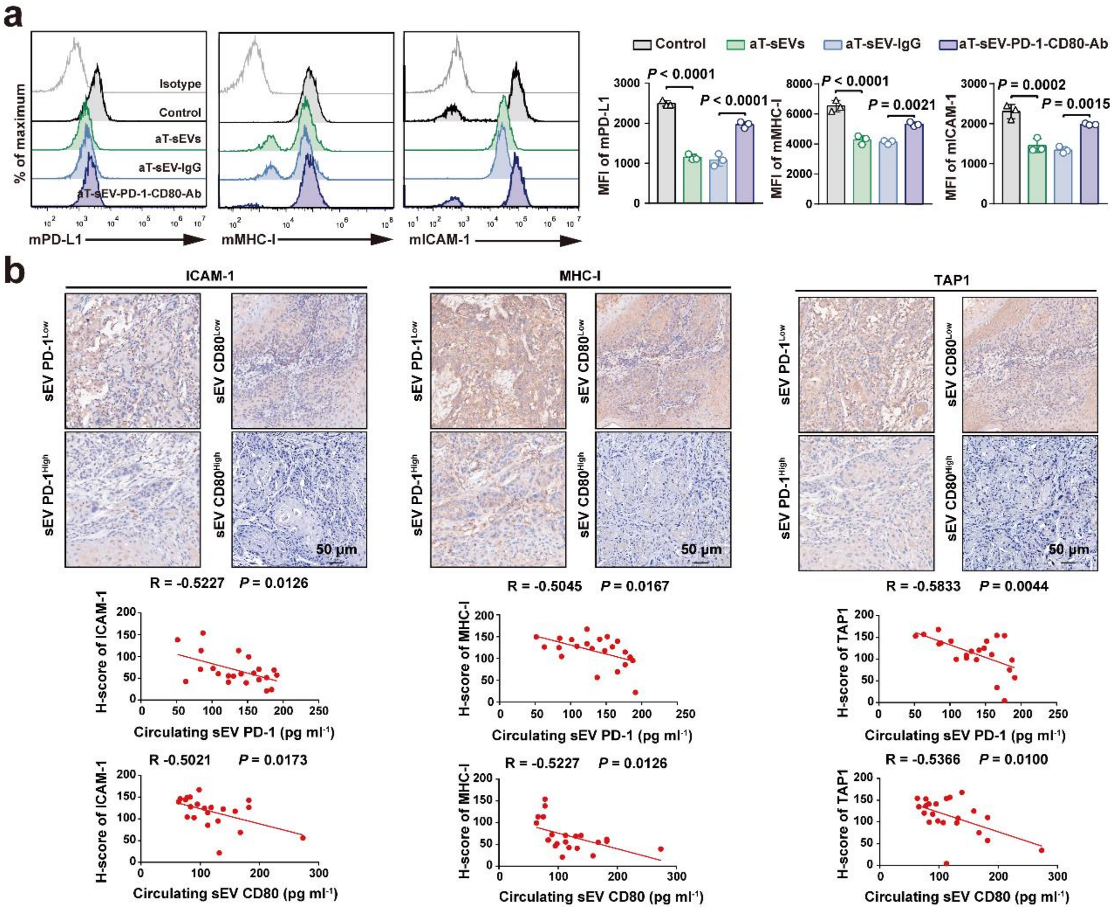

Supplementary Fig. 7 The level of circulating PD-1/CD80 were negatively correlated with molecular adhesion and immunogenicity in tumour cells

a Representative flow cytometric histograms (left) and quantification analysis (right) of the expression levels of mPD-L1, mMHC-I, and mICAM-1 on CAL27 cells after with indicated treatments. **b** Representative immunohistochemical images (top) of the expression of ICAM-1, MHC-I, and TAP in biopsies of HNSCC patients. Pearson correlation analysis of the intensity of ICAM-1, MHC-I and TAP with the circulating level of sEV PD-1/CD80 was performed in HNSCC patients with PD-L1$^+$ tumour cells (n = 22) (bottom). For **a** data were presented as mean ± S.D.; n = 3 biologically independent samples; One-way ANOVA. Source data are provided as a Source Data file.

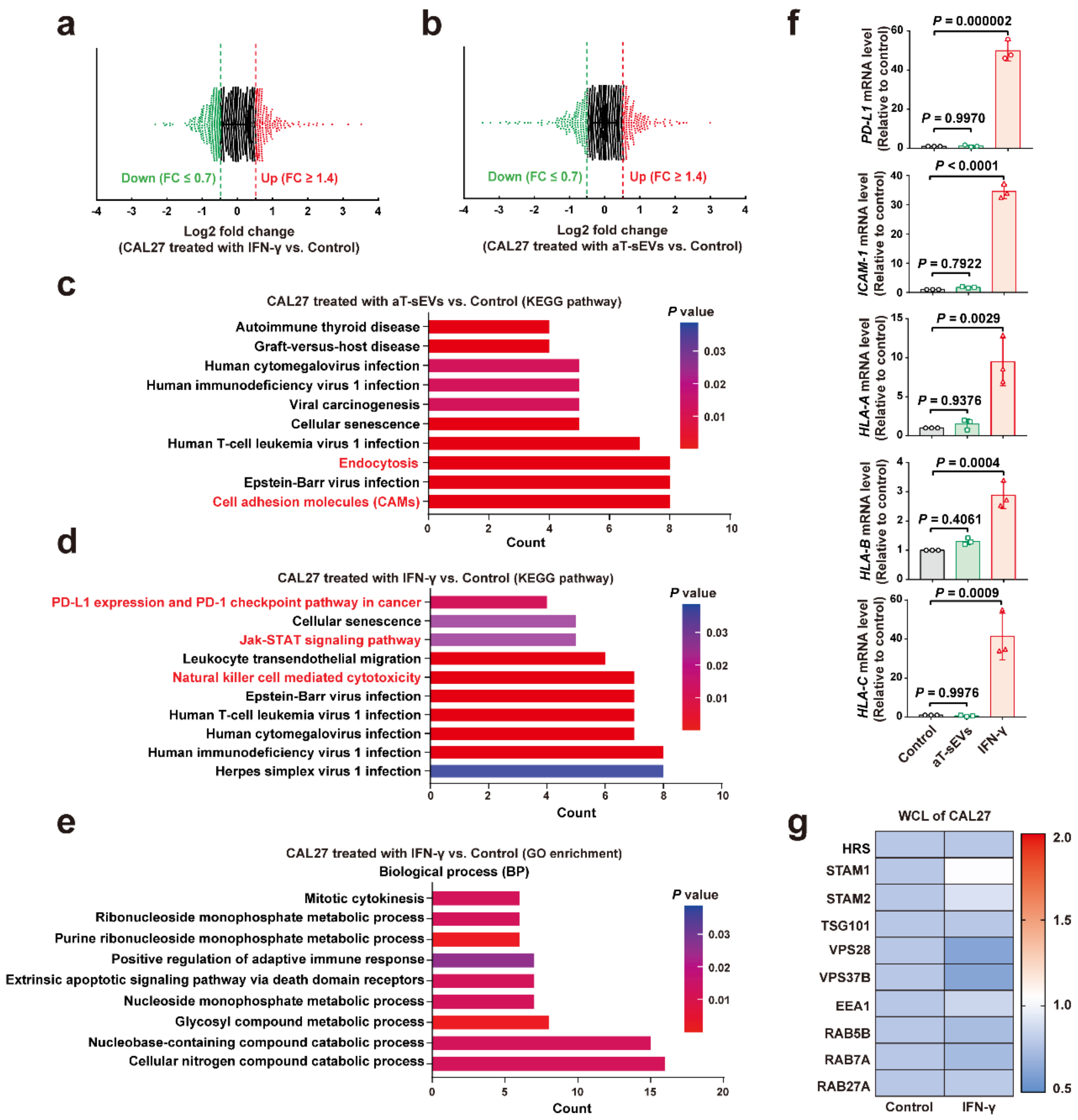

Supplementary Fig. 8 PD-1/CD80 on I-sEVs rather than IFN-γ initiated the ESCRT machinery in tumour cells

a-b MaxQuant analysis of the differential proteins in CAL27 cells treated with IFN-γ (**a**) or aT-sEV (**b**). **c-d** KEGG pathways analysis of DEGs in aT-sEV-treated (**c**) or IFN-γ-treated (**d**) CAL27 cells. KEGG, Kyoto encyclopedia of genes and genomes. **e** GO enrichment analysis of DEGs in CAL27 cells after treatment of IFN-γ. **f** Quantification analysis of the relative mRNA level of *PD-L1*, *ICAM-1*, *HLA-A*, *HLA-B*, and *HLA-C* in CAL27 cells with or without aT-sEV or IFN-γ treatment. **g** Heat map showing relative level of HRS, STAM-1, STAM-2, TSG101, VPS28, VPS37B, EEA1, RAB5B, RAB7A, and RAB27A in CAL27

cells after with IFN-γ treatment. For **f** data were presented as mean ± S.D.; n = 3 biologically independent samples; One-way ANOVA. The relevant raw data are provided as a Source Data file.

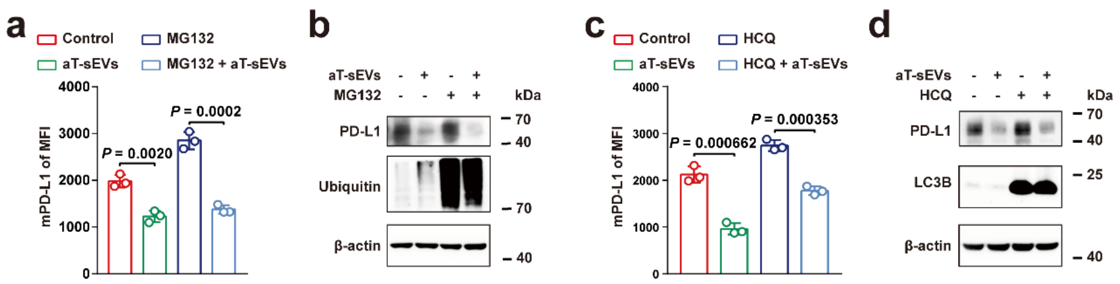

Supplementary Fig. 9 aT-sEVs had limited influence on degradation of PD-L1 protein in tumour cells

a-b Quantification (**a**) and western blotting (**b**) of the expression level of PD-L1 in aT-sEV-treated CAL27 cells with or without treatment of MG132. **c-d** Quantification (**c**) and western blotting (**d**) of the expression level of PD-L1 in aT-sEV-treated CAL27 cells with or without treatment of HCQ. For **a** and **c** data were presented as mean ± S.D.; n = 3 biologically independent samples; Two-sided *t*-test. The relevant raw data and uncropped blots are provided as a Source Data file.

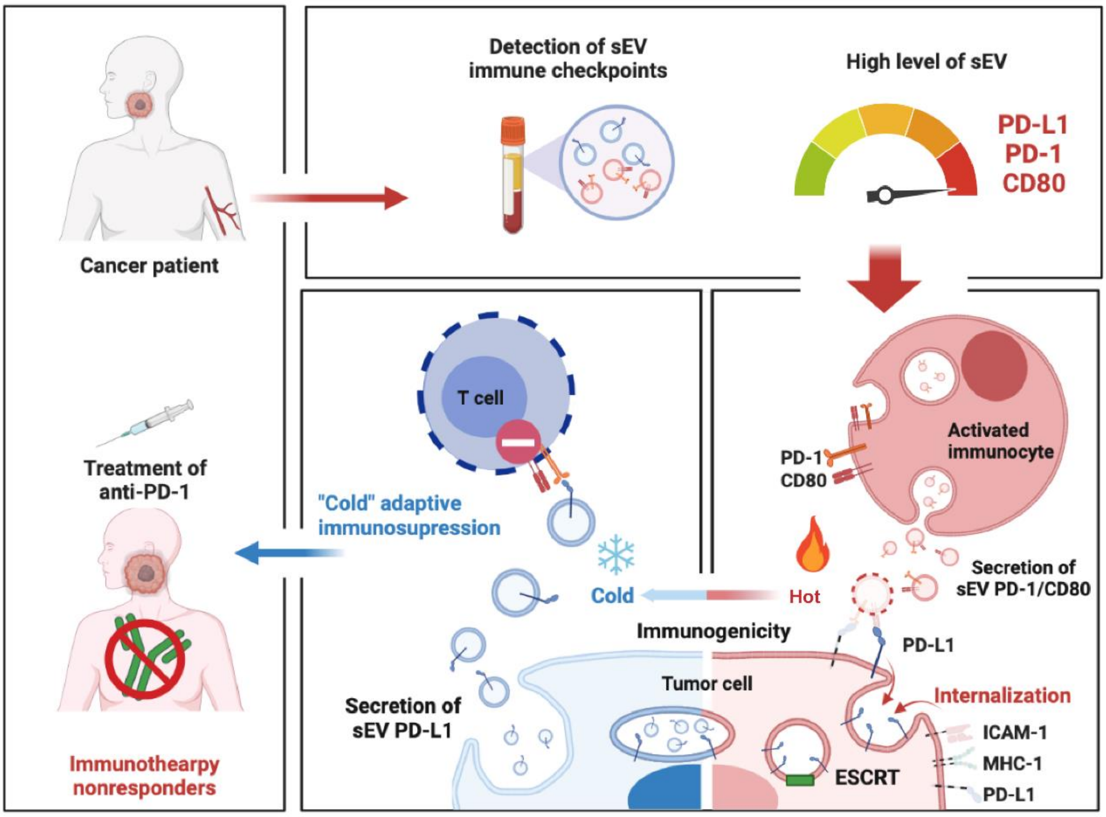

Supplementary Fig. 10 schematic of PD-1/CD80 sEVs inducing cold tumours featured with enhanced adaptive immunosuppression Immunocyte-derived sEVs (I-sEVs) carrying PD-1/CD80 increase secretion of sEV PD-L1 into circulation, and decreases membrane expression of PD-L1, as well as antigen presentation and intercellular adhesion molecules, on tumour cells, leading to an immunologically “cold” phenotype.

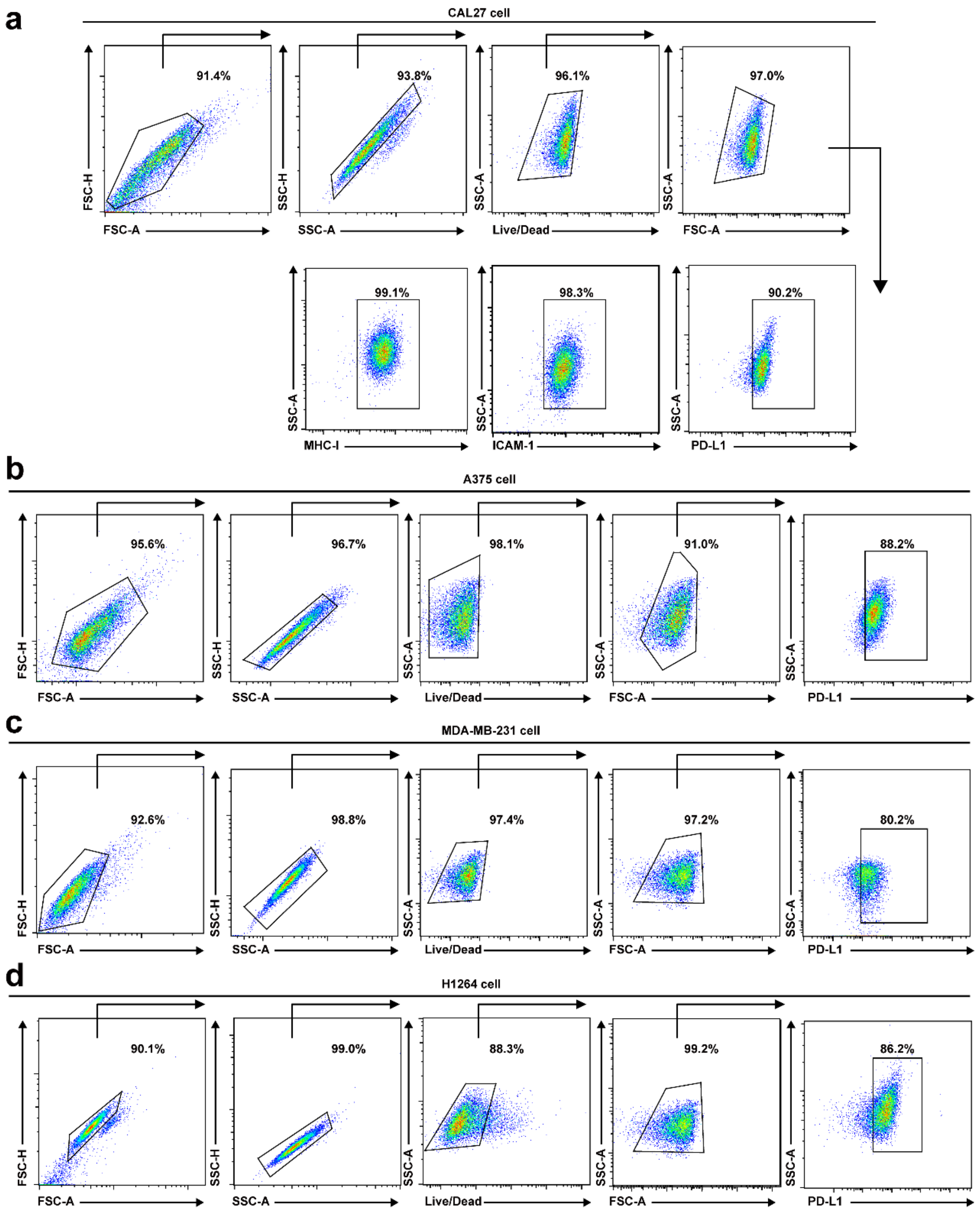

Supplementary Fig. 11 Gating strategies for flow cytometry analysis of CAL27 cells (a), A375 cells (b), MDA-MB-231 cells (c), and H1264 cells (d).

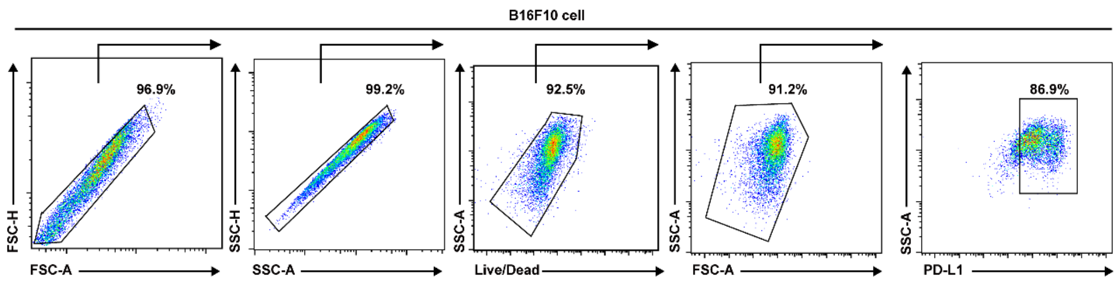

Supplementary Fig. 12 Gating strategy for B16F10 cells in flow cytometry analysis.

Supplementary tables
Supplementary Table 1

Summary of clinical features of HNSCC patients

Sample ID	Gender	Location	TNM stage	Follow up (months)	Survival	Anti-PD-1 therapy	Response*
1	Male	Tongue	T2N0M0	37	1	No	NA
2	Male	Tongue	T3N0M0	6	0	No	NA
3	Male	Cheek	T2N0M0	12	0	No	NA
4	Male	Cheek	T2N2M0	16	0	No	NA
5	Male	Cheek	T3N0M0	24	0	No	NA
6	Male	Tongue	T3N1M0	30	0	No	NA
7	Male	Cheek	T3N0M0	26	0	No	NA
8	Male	Tongue	T3N0M0	32	0	No	NA
9	Male	Mouth floor	T2N0M0	18	0	No	NA
10	Female	Gingiva	T4N2bM0	5	1	No	NA
11	Male	Tongue	T2N0M0	28	0	No	NA
12	Male	Cheek	T2N2M0	18	1	No	NA
13	Male	Palate	T2N2bM0	17	1	No	NA
14	Female	Mouth floor	T4N1M0	8	1	No	NA
15	Male	Tongue	T4N0M0	24	1	No	NA
16	Male	Gingiva	T1N0M0	32	0	No	NA
17	Male	Tongue	T2N1M0	32	0	No	NA
18	Male	Cheek	T3N1M0	24	1	No	NA
19	Female	Lip	T2N0M0	32	0	No	NA
20	Male	Tongue	T3N0M0	32	0	No	NA
21	Male	Tongue	T3N1M0	16	1	No	NA
22	Female	Tongue	T2N2M0	33	0	No	NA
23	Female	Gingiva	T3N2M0	32	0	No	NA
24	Male	Tongue	T4N1M0	16	1	No	NA
25	Male	Mouth floor	T3N0M0	34	0	No	NA
26	Female	Tongue	T2N0M0	34	0	No	NA
27	Male	Gingiva	T4N0M0	21	1	No	NA
28	Male	Mouth floor	T2N1M0	35	0	No	NA
29	Male	Cheek	T2N0M0	35	0	No	NA
30	Male	Tongue	T3N1M0	37	0	No	NA
31	Male	Tongue	T4N2M0	6	1	No	NA
32	Male	Tongue	T2N0M0	38	0	No	NA
33	Male	Gingiva	T4N2M0	8	1	No	NA
34	Male	Mouth floor	T3N1M0	10	1	No	NA
35	Male	Tongue	T1N1M0	38	0	No	NA
36	Male	Tongue	T2N0M0	38	0	No	NA

37	Male	Tongue	T3N3bM0	8	1	No	NA
38	Male	Cheek	T4aN0M0	12	1	No	NA
39	Male	Tongue	T3N2bM0	24	1	No	NA
40	Male	Gingiva	T4aN2bM0	32	1	No	NA
41	Female	Tongue	T1N0M0	43	0	No	NA
42	Female	Tongue	T2N1M0	14	1	No	NA
43	Male	Tongue	T2N2bM0	15	1	No	NA
44	Male	Cheek	T2N1M0	38	1	No	NA
45	Male	Tongue	T2N1M0	46	1	No	NA
46	Male	Tongue	T2N0M0	48	0	No	NA
47	Male	Gingiva	T1N0M0	36	0	No	NA
48	Male	Tongue	T2N0M0	38	1	No	NA
49	Male	Gingiva	T4aN1M0	14	1	No	NA
50	Male	Tongue	T3N2bM0	15	1	No	NA
51	Male	Tongue	T3N3bM0	24	1	No	NA
52	Male	Tongue	T3N2bM0	15	1	No	NA
53	Male	Gingiva	T1N2M0	18	1	Yes	SD
54	Male	Cheek	T4N3M0	5	1	Yes	PD
55	Male	Mouth floor	T2N0M0	6	1	Yes	PD
56	Male	Nasopharynx	T2N1M0	2	0	Yes	PR
57	Male	Nasopharynx	T1N3M1	8	0	Yes	PR
58	Male	Cheek	T4N2M0	13	1	Yes	PD
59	Male	Nasopharynx	T4N2M0	5	1	Yes	SD
60	Female	Cheek	T1N2M0	8	1	Yes	PD
61	Female	Palate	T2N2M0	8	0	Yes	PR
62	Male	Tongue	T2N2M0	18	0	Yes	CR
63	Female	Tongue	T4N2M1	6	0	Yes	CR
64	Male	Gingiva	T4N0M0	5	1	Yes	CR
65	Male	Nasopharynx	T2N2M0	10	1	Yes	CR
66	Male	Gingiva	T1N2M0	17	0	Yes	PD
67	Male	Cheek	T4N2M0	6	0	Yes	PR
68	Male	Tongue	T0N3bM0	15	0	Yes	PR
69	Female	Cheek	T3N2bM0	7	0	Yes	SD
70	Male	Tongue	T0N3M0	2	1	Yes	SD
71	Male	Tongue	T2N2M0	3	1	Yes	SD
72	Female	Gingiva	T4N2MO	2	1	Yes	PD
73	Male	Tongue	T3N3M0	4	0	Yes	PR
74	Male	Tongue	T0N3M0	5	0	Yes	CR
75	Female	Cheek	T0N3bM0	3	0	Yes	CR

***, response was analyzed by irRECIST. CR, complete response. PR, partial response. SD, stable disease. PD, progressive disease; all patients were over 18 years old.**

Supplementary Table 2

A list of reagents used in this study		
**REAGENT or RESOURCE**	**SOURCE**	**IDENTIFIER**
**Antibodies**		
Immunoblotting: Anti-human PD-L1 (E1L3N)	Cell Signaling Technology	Cat. 13684
Immunoblotting: Anti-human PD-1 (D4W2J)	Cell Signaling Technology	Cat. 86163
Immunoblotting: Anti-human CD80 (E3Q9V)	Cell Signaling Technology	Cat. 15416
Immunoblotting: Anti-human CD3ε (D7A6E™)	Cell Signaling Technology	Cat. 85061
Immunoblotting: Anti-human/mouse TSG101 (4A10)	Abcam	Cat. ab83
Immunoblotting: Anti-human CD81 (D3N2D)	Cell Signaling Technology	Cat. 56039
Immunoblotting: Anti-human/mouse HRS (D7T5N)	Cell Signaling Technology	Cat. 15087
Immunoblotting: Anti-human/mouse ALIX (3A9)	Biolegend	Cat. 634502
Immunoblotting: Anti-Ubiquitin (P4D1)	Cell Signaling Technology	Cat. 3936
Immunoblotting: Anti-human LC3B (D11)	Cell Signaling Technology	Cat. 3868
Immunoblotting: Anti-human/mouse GAPDH	ABclonal	Cat. AC033
Immunoblotting: Anti-human/mouse β-Actin	ABclonal	Cat. AC206
Nanoparticle flow cytometry (Nano-FCM): Anti-human CD45 PE-Cy7 (HI30)	BD Biosciences	Cat. 557748
Nano-FCM: Anti-human EpCAM FITC (EBA1)	BD Biosciences	Cat. 347197
Nano-FCM: Anti-human CD144 BV421 (55-7H1)	BD Biosciences	Cat. 565670
Nano-FCM: Anti-human PD-1 PE (EH12.2H7)	Biolegend	Cat. 329906
Nano-FCM: Anti-human CD80 (B7-1) PE (2D10)	Biolegend	Cat. 305208
Nano-FCM: Anti-human CD80 (B7-1) BV421 (2D10)	Biolegend	Cat. 305222
Nano-FCM: Anti-human PD-L1 PE (9E.2A3)	Biolegend	Cat. 329706
Nano-FCM: Anti-human PD-L1 APC (9E.2A3)	Biolegend	Cat. 329708
Nano-FCM: Anti-human ICAM-1 FITC (HA58)	Biolegend	Cat. 353107
Nano-FCM: Anti-human CD47 APC (CC2C6)	Biolegend	Cat. 323124
Nano-FCM: Anti-human EGFR PE (AY13)	Biolegend	Cat. 352906
Nano-FCM: Anti-human CD86 PE (IT2.2)	Biolegend	Cat. 305406
Nano-FCM: Anti-human CTLA-4 PerCP/Cy5.5 (BNI3)	Biolegend	Cat. 369608
Nano-FCM: Anti-human LAG-3 AF647 (T47-530)	BD Biosciences	Cat. 565716
Nano-FCM: Anti-human TIM-3 BV421 (7D3)	BD Biosciences	Cat. T47-530
Nano-FCM: Anti-human CD3 FITC (OKT3)	Biolegend	Cat. 317306
Nano-FCM: Anti-human CD4 PE-Cy7 (OKT4)	Biolegend	Cat. 317414
Nano-FCM: Anti-human CD8 APC (SK1)	Biolegend	Cat. 344722
Nano-FCM: Anti-human CD11c PerCP (Bu15)	Biolegend	Cat. 337234
Nano-FCM: Anti-human CD19 PE-Cy7 (HIB19)	Biolegend	Cat. 302216
Nano-FCM: Anti-human CD68 APC (Y1/82A)	Biolegend	Cat. 333810
Nano-FCM: Anti- mouse PD-L1 APC (10F.9G2)	Biolegend	Cat. 124312
Flow cytometry: Anti-human PD-L1 APC (9E.2A3)	Biolegend	Cat. 329708
Flow cytometry: Anti-human PD-L1 PE (9E.2A3)	Biolegend	Cat. 329706

Flow cytometry: Anti-human HLA-A, B, C APC (W6/32)	Biolegend	Cat. 311410
Flow cytometry: Anti-human ICAM-1 FITC (HA58)	Biolegend	Cat. 353107
Flow cytometry: Anti-mouse PD-L1 APC (10F.9G2)	Biolegend	Cat. 124312
Immunogold labelling: Anti-human PD-1	R&D System	Cat. DY1086
Immunogold labelling: Anti-human CD80	R&D System	Cat. DY140
Blocking: Anti-human PD-1 (Pembrolizumab)	BioXCell	Cat. SIM0010
Blocking: Anti-human PD-L1 (MIH1)	eBioscience	Cat. 13-5983-80
Blocking: Anti-human CD80 (W17149G)	Biolegend	Cat. 621803
Blocking: Anti- mouse PD-1 (RMP1-14)	BioXCell	Cat. BE0146
Blocking: Anti- mouse CD80 (16-10A1)	eBioscience	Cat. 16-0801-82
Blocking: Mouse IgG isotype (MOPC-21)	BioXCell	Cat. BE0083
Blocking: Rat IgG2b isotype (LTF-2)	BioXCell	Cat. BE0090
ELISA: Human IFN-γ	Mabtech	Cat. 3420-1HP-1
ELISA: Human PD-1	R&D System	Cat. DY1086
ELISA: Human CD80	R&D System	Cat. DY140
ELISA: Anti-human PD-L1 (5H1) (capture)	Millipore	Cat. MABC1115
ELISA: Anti-human PD-L1 (biotin) (MIH1)	eBioscience	Cat. 13-5983-82
Immunofluorescence: Anti-human PD-L1 (5H1)	Millipore	Cat. MABC1115
Immunofluorescence (FRET): Anti-human PD-L1 (28-8)	Abcam	Cat. ab205921
Immunofluorescence (FRET): Anti-human PD-1 (NAT105)	Abcam	Cat. ab52587
Immunofluorescence: Anti-human CD63 (MEM-259)	Abcam	Cat. ab8219
Immunofluorescence: Anti-human/mouse HRS (D7T5N)	Cell Signaling Technology	Cat. 15087S
Immunofluorescence: Anti-human/mouse EEA1 (1G11)	eBioscience	Cat. 14-9114-82
Immunofluorescence: Anti-human/mouse RAB7 (E907E)	Cell Signaling Technology	Cat. 95746
Immunofluorescence: Anti-mouse CD8α (D4W2Z)	Cell Signaling Technology	Cat. 98941T
Immunohistochemical: Anti-human PD-L1 (405.9A11)	Cell Signaling Technology	Cat. 29122
Immunohistochemical: Anti-human ICAM-1 (EPR24639-3)	Abcam	Cat. ab222736
Immunohistochemical: Anti-human HLA Class I ABC (EPR22172)	Abcam	Cat. ab225636
Immunohistochemical: Anti- human/mouse TAP	Proteintech	Cat. 11114-1-AP
Immunohistochemical: Anti-mouse CD8α (D4W2Z)	Cell Signaling Technology	Cat. 98941T
Immunohistochemical: Anti-mouse PD-L1 (D5V3B)	Cell Signaling Technology	Cat. 64988
**Chemicals, Peptides, and Recombinant Proteins**		
HCQ (Hydroxychloroquine)	MedChemExpress	Cat. HY-W031727
MG132	MedChemExpress	Cat. HY-13259
Human IFN-γ	PeproTech	Cat. 300-02
PhosSTOP	Sigma-Aldrich	Cat. 4906837001
Complete mini proteasome inhibitors	Sigma-Aldrich	Cat. 05892791001
**Critical Commercial Assays**		
HiScript II Q RT SuperMix for qPCR (+gDNA wiper)	Vazyme	Cat. R223-01
ChamQ SYBR qPCR Master Mix	Vazyme	Cat. Q311-02/03
