# Supplementary material for: PD-1/CD80+ small extracellular vesicles from immunocytes induce cold tumours featured with enhanced adaptive immunosuppression
Source: Nat Commun. 2024 May 8;15:3884. doi: 10.1038/s41467-024-48200-9 (PMC11079016; doi:10.1038/s41467-024-48200-9)
Supplement: Supplementary file 3 — Reporting Summary [file 41467_2024_48200_MOESM3_ESM.pdf]

nature portfolio

Corresponding author(s): Gang Chen

Last updated by author(s): Apr 18, 2024

Reporting Summary

Nature Portfolio wishes to improve the reproducibility of the work that we publish. This form provides structure for consistency and transparency in reporting. For further information on Nature Portfolio policies, see our Editorial Policies and the Editorial Policy Checklist.

Statistics

For all statistical analyses, confirm that the following items are present in the figure legend, table legend, main text, or Methods section.

n/a	Confirmed	
☐	☒	The exact sample size (*n*) for each experimental group/condition, given as a discrete number and unit of measurement
☐	☒	A statement on whether measurements were taken from distinct samples or whether the same sample was measured repeatedly
☐	☒	The statistical test(s) used AND whether they are one- or two-sided<br>*Only common tests should be described solely by name; describe more complex techniques in the Methods section.*
☒	☐	A description of all covariates tested
☒	☐	A description of any assumptions or corrections, such as tests of normality and adjustment for multiple comparisons
☐	☒	A full description of the statistical parameters including central tendency (e.g. means) or other basic estimates (e.g. regression coefficient) AND variation (e.g. standard deviation) or associated estimates of uncertainty (e.g. confidence intervals)
☐	☒	For null hypothesis testing, the test statistic (e.g. *F*, *t*, *r*) with confidence intervals, effect sizes, degrees of freedom and *P* value noted<br>*Give P values as exact values whenever suitable.*
☒	☐	For Bayesian analysis, information on the choice of priors and Markov chain Monte Carlo settings
☒	☐	For hierarchical and complex designs, identification of the appropriate level for tests and full reporting of outcomes
☐	☒	Estimates of effect sizes (e.g. Cohen's *d*, Pearson's *r*), indicating how they were calculated

Our web collection on statistics for biologists contains articles on many of the points above.

Software and code

Policy information about availability of computer code

Data collection	A50 micro plus Flow Cytometry for nanoparticle flow cytometry ;<br>CFX96 Real-Time PCR Detection System for RT-qPCR;<br>PEAKS Studio X+ software for mass spectrometry-based proteomics data analysis;<br>CytoFLEX for flow cytometry;<br>ZetaView for nanoparticle tracking analysis.
Data analysis	GraphPad Prism V9.3.1 for graphs and statistical analysis;<br>FlowJo 10.4.2 for FACS plots;<br>Custer 3.0 for hierarchical analysis;<br>ImageJ 1.52 for immunohistochemistry;<br>Software R 2.14 for Random Forest.

For manuscripts utilizing custom algorithms or software that are central to the research but not yet described in published literature, software must be made available to editors and reviewers. We strongly encourage code deposition in a community repository (e.g. GitHub). See the Nature Portfolio guidelines for submitting code & software for further information.

Data

Policy information about availability of data

All manuscripts must include a data availability statement. This statement should provide the following information, where applicable:

- Accession codes, unique identifiers, or web links for publicly available datasets
- A description of any restrictions on data availability
- For clinical datasets or third party data, please ensure that the statement adheres to our policy

The authors declare that the data supporting the findings of this study are available within the paper and its supplementary information files. Source data are provided with this paper.

Research involving human participants, their data, or biological material

Policy information about studies with human participants or human data. See also policy information about sex, gender (identity/presentation), and sexual orientation and race, ethnicity and racism.

Reporting on sex and gender	There was no restriction on gender in our study. Both healthy donors and HNSCC patients were over 18 years old.
Reporting on race, ethnicity, or other socially relevant groupings	Our study has no socially relevant such as race and ethnicity or other socially relevant groupings.
Population characteristics	Patients' demographics and disease characteristics are summarized in Supplementary Table 1.
Recruitment	52 primary head and neck squamous cell carcinoma (HNSCC) patients and 23 recurrent or metastatic HNSCC patients treated with PD-1 antibody (anti-PD-1) were enrolled in this study.
Ethics oversight	The study was conducted strictly based on the guidelines setting forth by the Medical Ethics Committee of Hospital of Stomatology Wuhan University. All patients had signed the informed consents and blood samples from primary head and neck squamous cell carcinoma (HNSCC) patients used in this expriment were collected at the Department of Oral and Maxillofacial Surgery, School and Hospital of Stomatology Wuhan University. All participants received a compensation of transportation expanses.

Note that full information on the approval of the study protocol must also be provided in the manuscript.

Field-specific reporting

Please select the one below that is the best fit for your research. If you are not sure, read the appropriate sections before making your selection.

☒ Life sciences ☐ Behavioural & social sciences ☐ Ecological, evolutionary & environmental sciences

For a reference copy of the document with all sections, see nature.com/documents/nr-reporting-summary-flat.pdf

Life sciences study design

All studies must disclose on these points even when the disclosure is negative.

Sample size	Samples size for each experiment is indicated in the figures or corresponding figure legends. No statistical method was used to predetermine sample size because such sample sizes are sufficient for a statistical analysis based on similarly literature reporting (Chen et al., Nature, 2018, 560(7718):382-386; Gao et al., Nat Cell Biol. 2020 Sep;22(9):1064-1075.). For in vitro experiments including RT-qPCR assays, three biologically independent experiments were conducted unless otherwise stated. For in vivo mice experiments, a sample size of n ≥ 4 mice per group was sufficient for statistical analysis and generating significant results based previous studies (Wang et al., Nature, 2017; 546(7658):426-430; Simoneschi et al., Nature, 2021; 592(7856):789-793.)
Data exclusions	No sample was excluded in our analysis.
Replication	All replicates reported in the manuscript are biological replicates. All the statistics reported in the manuscript are based on at least 3 biologically independent replicates. All attempts to replicate the experiments were successful.
Randomization	All samples used in this study were randomly allocated into different experimental groups.
Blinding	The assessment of clinical responses for patients was performed independently in a double-blind fashion. For mice studies, the experiments were performed in a blinded fashion when possible. The investigators were not blinded to sample allocation during experiments because the information of the value of tumor size and different treatments among groups was essential and correctly to conduct the studies. Downstream analyses of mouse samples (immunofluorescence staining, flow cytometry and ELISA) were performed in a blinded fashion, which means that people performing the assays were not aware of the treatment groups until the data analyses were completed.

Reporting for specific materials, systems and methods

We require information from authors about some types of materials, experimental systems and methods used in many studies. Here, indicate whether each material, system or method listed is relevant to your study. If you are not sure if a list item applies to your research, read the appropriate section before selecting a response.

Materials & experimental systems

n/a	Involved in the study
☐	☒ Antibodies
☐	☒ Eukaryotic cell lines
☒	☐ Palaeontology and archaeology
☐	☒ Animals and other organisms
☒	☐ Clinical data
☒	☐ Dual use research of concern
☒	☐ Plants

Methods

n/a	Involved in the study
☒	☐ ChIP-seq
☐	☒ Flow cytometry
☒	☐ MRI-based neuroimaging

Antibodies

Antibodies used

All primary antibodies used in this study were shown in Supplementary Table 2.

The following primary antibodies were used for western blotting (Immunoblotting). They are listed as antigen first, followed by dilution, host, supplier, catalog number and clone/lot number as applicable.
1) Anti-human PD-L1, 1:500, Rabbit, Cell Signaling Technology, #13684, clone E1L3N;
2) Anti-PD-1, 1:1000, Rabbit, Cell Signaling Technology, #86163, clone D4W2J;
3) Anti-human CD80, 1:1000, Rabbit, Cell Signaling Technology, #15416, clone E3Q9V;
4) Anti-human CD3ε, 1:1000, Rabbit, Cell Signaling Technology, #85061, clone D7A6E™;
5) Anti-human/mouse TSG101, 1:1000, Rabbit, Abcam, #ab83, clone 4A10;
6) Anti-human CD81, 1:1000, Rabbit, Cell Signaling Technology, #56039, clone D3N2D;
7) Anti-human/mouse HRS, 1:1000, Rabbit, Cell Signaling Technology, #15087, clone D7T5N8;
8) Anti-human/mouse ALIX, 1:500, Mouse, Biolegend, #634502, clone 3A9;
9) Anti-Ubiquitin, 1:1000, Rabbit, Cell Signaling Technology, #3936, clone P4D1;
10) Anti-human LC38 , 1:1000, Rabbit, Cell Signaling Technology, #3868, clone D11;
11) Anti-GAPDH, 1:10000, Mouse, ABclonal, #AC033;
12) Anti-β-Actin, 1:10000, Rabbit, ABclonal, #AC206.

The following primary antibodies were used for nanoparticle flow cytometry (Nano-FCM). They are listed as antigen first, followed by dilution, host, supplier, catalog number and clone/lot number as applicable.
1) Anti-human EpCAM (FITC), 1:10, Mouse, BD Biosciences, #347197, clone EBA1;
2) Anti-human CD144 BV421, 1:10, Mouse, BD Biosciences, #565670, clone 55-7H1;
3) Anti-human CD45 PE-Cy7, 1:10, Mouse, BD Biosciences, #557748, clone HI30;
4) Anti-human PD-1 (PE), 1:10, Mouse, BioLegend, #329906, clone EH12.2H7;
5) Anti-human CD80 (PE), 1:10, Mouse, BioLegend, #305208, clone 2D10;
6) Anti-human CD80 (BV421), 1:10, Mouse, BioLegend, #305222, clone 2D10;
7) Anti-human PD-L1 (PE), 1:10, Mouse, BioLegend, #329706, clone 9E.2A3;;
8) Anti-human PD-L1 (APC), 1:10, Mouse, BioLegend, #329708, clone 9E.2A3;
9) Anti-human ICAM-1 (FITC), 1:10, Mouse, BioLegend, #353107, clone HA58;
10) Anti-human CD47 (APC), 1:10, Mouse, BioLegend, #323124, clone CC2C6;
11) Anti-human EGFR (PE), 1:10, Mouse, BioLegend, #352906, clone AY13;
12) Anti-human CD86 (PE), 1:10, Mouse, BioLegend, #305406, clone IT2.2;
13) Anti-human CTLA-4 (PerCP/Cy5.5), 1:10, Mouse, BioLegend, #369608, clone BNI3;
14) Anti-human LAG-3 (AF647), 1:10, Mouse, BD Biosciences, #565716, clone T47-530;
15) Anti-human TIM-3 (BV421), 1:10, Mouse, BD Biosciences, #565562, clone 7D3;
16) Anti-human CD3 (FITC), 1:10, Mouse, BioLegend, #317306, clone OKT3;
17) Anti-human CD4 (PE-Cy7), 1:10, Mouse, BioLegend, #317414, clone OKT4;
18) Anti-human CD8 (APC), 1:10, Mouse, BioLegend, #344722, clone SK1;
19) Anti-human CD11c (PerCP), 1:10, Mouse, BioLegend, #337234, clone Bu15;
20) Anti-human CD19 (PE-Cy7), 1:10, Mouse, BioLegend, #302216, clone HIB 19;
21) Anti-human CD68 (APC), 1:10, Mouse, Biolegend, #333810, clone Y1/82A;
22) Anti- mouse PD-L1 (APC), 1:10, Mouse, Biolegend, #124312, clone 10F.9G2.

The following primary antibodies were used for flow cytometry. They are listed as antigen first, followed by dilution, host, supplier, catalog number and clone/lot number as applicable.
1) Anti-human PD-L1 (APC), 1:50, Mouse, BioLegend, #329708, clone 9E.2A3;
2) Anti-human PD-L1 (PE), 1:50, Mouse, BioLegend, #329706, clone 9E.2A3;
3) Anti-human HLA-A, B, C (APC), 1:50, Mouse, BioLegend, #311410, clone W6/32;
4) Anti-human ICAM-1 (FITC), 1:50, Mouse, BioLegend, #353107, clone HA58;
5) Anti-mouse PD-L1 (APC), 1:50, Rat, BioLegend, #124312, clone 10F.9G2.

The following primary antibodies were used for blocking. They are listed as antigen first, followed by dilution, host, supplier, catalog number and clone/lot number as applicable.
1) Anti-human PD-1 (Pembrolizumab), 1:100, Mouse, Bio X Cell, #SIM0010;
2) Anti-human PD-L1, 1:100, Mouse, eBioscience, #13-5983-80; clone MIH1;
3) Anti-human CD80, 1:100, Rat, Biolegend, #621803, clone W17149G;
4) Anti-mouse PD-1, 1:100, Rat, Bio X Cell, #BE0146, clone RMP1-14;
5) Anti-mouse CD80, 1:100, Rat, eBioscience, #16-0801-82, clone 16-10A1;
6) Mouse IgG isotype, 1:100, Mouse, Bio X Cell, #BE0083, clone MOPC-21;
7) Rat IgG2b isotype control, 1:600, Rat, Bio X Cell, #BE0090, clone LTF-2.

The following primary antibodies were used for ELISA. They are listed as antigen first, followed by dilution, host, supplier, catalog number and clone/lot number as applicable.
1) For capture, anti-human PD-L1, 1:200, Mouse, Millipore, clone 5H1;
2) For detection, anti-human PD-L1 (biotin), 1:500, Mouse, eBioscience, #13-5983-82, clone MIH1;
3) Human B7-1/CD80 DuoSet ELISA (capture antibody: 1:125, detection antibody: 1:4000), R&D Systems, #DY140;
4) Human PD-1 DuoSet ELISA (capture antibody: 1:500, detection antibody: 1:250), R&D Systems, #DY1086;
5) Human IFN-γ (capture antibody: 1:500, detection antibody: 1:1000), Mabtech, #3420-1HP-1.

The following primary antibodies were used for immunofluorescence/immunohistochemistry. They are listed as antigen first, followed by dilution, host,
supplier, catalog number and clone/lot number as applicable.
1) Anti-human PD-L1, 1:200, Mouse, Millipore, clone 5H1;
2) Anti-human PD-L1, 1:200, Rabbit, Cell Signaling Technology, #86744, clone D8T4X;
3) Anti-human CD63, 1:100, Mouse, Abcam, ab8219, clone MEM-259;
4) Anti-Hrs, 1:200, Rabbit, Cell Signaling Technology, #15087S, clone D7T5N;
5) Anti-mouse CD8a, 1:200, Cell Signaling Technology, #98941T, clone D4W2Z;
6) Anti-human/mouse EEA1 (1G11), 1:200, eBioscience, #14-9114-82, clone 1G11;
7) Anti-human/mouse RAB7, 1:200, Cell Signaling Technology, #95746, clone E907E;
8) Anti-human PD-L1, 1:200, Cell Signaling Technology, #29122, clone E405.9A11;
9) Anti-human ICAM-1, 1:200, Abcam, #ab222736, clone EPR24639-3;
10) Anti-human HLA Class I ABC, 1:200, Abcam, #ab225636, clone EPR22172;
11) Anti- human/mouse TAP, 1:200, Proteintech, #11114-1-AP;
12) Anti-mouse PD-L1, 1:200, Cell Signaling Technology, #64988, clone D5V3B.

Validation

The following antibodies used in our study have been validated and detailed information could be found on the website from manufactures as listed below:
1) Anti-human PD-L1, https://www.cellsignal.cn/products/primary-antibodies/pd-l1-e1l3n-xp-rabbit-mab/13684?_=1613959174547&Ntt=13684&tahead=true;
2) Anti-human PD-1, https://www.cellsignal.cn/products/primary-antibodies/pd-1-intracellular-domain-d4w2j-xp-174-rabbit-mab/86163;
3) Anti-human CD80, https://www.cellsignal.cn/products/primary-antibodies/cd80-e3q9v-rabbit-mab/15416;
4) Anti-human CD3ε, https://www.cellsignal.cn/products/primary-antibodies/cd3e-d7a6e-8482-xp-174-rabbit-mab/85061;
5) Anti-human/mouse TSG101, https://www.abcam.cn/products/primary-antibodies/tsg101-antibody-4a10-bsa-and-azide-free-ab83.html;
6) Anti-human CD81, https://www.cellsignal.cn/products/primary-antibodies/cd81-d3n2d-rabbit-mab/56039;
7) Anti-human/mouse HRS, https://www.cellsignal.cn/products/primary-antibodies/hrs-d7t5n-rabbit-mab/15087;
8) Anti-human/mouse ALIX, https://www.biolegend.com/en-gb/products/purified-anti-alix-antibody-4469;
9) Anti-Ubiquitin, https://www.cellsignal.cn/products/primary-antibodies/ubiquitin-p4d1-mouse-mab/3936;
10) Anti-human LC3B, https://www.cellsignal.cn/products/primary-antibodies/lc3b-d11-xp-174-rabbit-mab/3868;
11) Anti-human/mouse GAPDH, https://abclonal.com.cn/catalog/AC033;
12) Anti-human/mouse β-Actin, https://abclonal.com.cn/catalog/AC206;
13) Anti-human CD45 (PE-Cy7), https://www.bdbiosciences.com/en-us/search-results?searchKey=557748;
14) Anti-human EpCAM (FITC), https://www.bdbiosciences.com/en-us/search-results?searchKey=347197;
15) Anti-human CD144 (BV421), https://www.bdbiosciences.com/en-us/search-results?searchKey=565670;
16) Anti-human PD-1 (PE), https://www.biolegend.com/en-gb/products/pe-anti-human-cd279-pd-1-antibody-4412;
17) Anti-human CD80 (PE), https://www.biolegend.com/en-gb/products/pe-anti-human-cd80-antibody-554;
18) Anti-human PD-L1 (PE), https://www.biolegend.com/en-gb/products/pe-anti-human-cd274-b7-h1-pd-l1-antibody-4375;
19) Anti-human PD-L1 (APC), https://www.biolegend.com/en-gb/products/apc-anti-human-cd274-b7-h1-pd-l1-antibody-4376;
20) Anti-human ICAM-1 (FITC), https://www.biolegend.com/en-gb/products/fitc-anti-human-cd54-antibody-7448;
21) Anti-human CD47 (APC), https://www.biolegend.com/en-gb/products/apc-anti-human-cd47-antibody-14976;
22) Anti-human EGFR (PE), https://www.biolegend.com/en-gb/products/apc-anti-human-egfr-antibody-7714;
23) Anti-human CD86 (PE), https://www.biolegend.com/en-gb/products/pe-anti-human-cd86-antibody-778;
24) Anti-human CTLA-4 (PerCP/Cy5.5), https://www.biolegend.com/en-gb/products/percp-cyanine5-5-anti-human-cd152-ctla-4-antibody-13584;
25) Anti-human LAG-3 (AF647), https://www.bdbiosciences.com/en-us/search-results?searchKey=565716;
26) Anti-human TIM-3 (BV421), https://www.bdbiosciences.com/en-us/search-results?searchKey=T47-530;
27) Anti-human CD3 (FITC), https://www.biolegend.com/en-gb/products/fitc-anti-human-cd3-antibody-3644;
28) Anti-human CD4 (PE-Cy7), https://www.biolegend.com/en-gb/products/pe-cyanine7-anti-human-cd4-antibody-3656;
29) Anti-human CD8 (APC), https://www.biolegend.com/en-gb/products/apc-anti-human-cd8-antibody-6531;
30) Anti-human CD11c (PerCP), https://www.biolegend.com/en-gb/products/percp-anti-human-cd11c-antibody-12734;
31) Anti-human CD19 (PE-Cy7), https://www.biolegend.com/en-gb/products/pe-cyanine7-anti-human-cd19-antibody-1911;
32) Anti-human CD68 (APC), https://www.biolegend.com/en-gb/products/apc-anti-human-cd68-antibody-6542;
33) Anti- mouse PD-L1 (APC), https://www.biolegend.com/en-gb/products/apc-anti-mouse-cd274-b7-h1-pd-l1-antibody-6655;
34) Anti-human HLA-A, B, C (APC), https://www.biolegend.com/en-gb/products/apc-anti-human-hla-a-b-c-antibody-1870;
35) Anti-human PD-1, https://www.bioxcell.com.cn/product/catalogsearch.html?q=SIM0010;
36) Anti-human PD-L1, https://www.thermofisher.cn/cn/zh/antibody/product/CD274-PD-L1-B7-H1-Antibody-clone-MIH1-

Monoclonal/13-5983-80
37) Anti-human CD80, https://www.biolegend.com/en-gb/products/ultra-leaf-purified-anti-human-cd80-antibody-18531;
38) Anti- mouse PD-1, https://www.bioxcell.com.cn/product/catalogsearch.html?q=BE0146;
39) Anti- mouse CD80, https://www.thermofisher.cn/cn/zh/antibody/product/CD80-B7-1-Antibody-clone-16-10A1-Monoclonal/16-0801-82;
40) Mouse IgG isotype, https://www.bioxcell.com.cn/product/catalogsearch.html?q=BE0083;
41) Rat IgG2b isotype, https://www.bioxcell.com.cn/product/catalogsearch.html?q=BE0090;
42) Human IFN-γ, https://www.mabtech.com/products?q=3420-1HP-1;
43) Human PD-1, https://www.rndsystems.com/cn/products/human-pd-1-duoset-elisa_dy1086;
44) Human CD80, https://www.rndsystems.com/cn/products/human-b7-1-cd80-duoset-elisa_dy140;
45) Anti-human PD-L1, https://www.sigmaaldrich.cn/CN/zh/search/mabc1115?focus=products&page=1&perpage=30&sort=relevance&term=MABC1115&type=product;
46) Anti-human PD-L1 (biotin), https://www.thermofisher.cn/cn/zh/antibody/product/CD274-PD-L1-B7-H1-Antibody-clone-MIH1-Monoclonal/13-5983-82;
47) Anti-human PD-L1, https://www.abcam.cn/products/primary-antibodies/pd-l1-antibody-28-8-ab205921.html;
48) Anti-human PD-1, https://www.abcam.cn/products/primary-antibodies/pd1-antibody-nat105-ab52587.html;
49) Anti-human CD63, https://www.abcam.cn/products/primary-antibodies/cd63-antibody-mem-259-ab8219.html;
50) Anti-human/mouse EEA1, https://www.thermofisher.cn/cn/zh/antibody/product/EEA1-Antibody-clone-1G11-Monoclonal/14-9114-82;
51) Anti-human/mouse RAB7, https://www.cellsignal.cn/products/primary-antibodies/rab7-e9o7e-mouse-mab/95746;
52) Anti-mouse CD8α, https://www.cellsignal.cn/products/primary-antibodies/cd8a-d4w2z-xp-rabbit-mab/98941;
53) Anti-human PD-L1, https://www.cellsignal.cn/products/primary-antibodies/pd-l1-405-9a11-mouse-mab/29122;
54) Anti-human ICAM-1, https://www.abcam.cn/products/primary-antibodies/icam1-antibody-epr22161-284-ab222736.html;
55) Anti-human HLA Class I ABC, https://www.abcam.cn/products/primary-antibodies/hla-class-1-abc-antibody-epr22172-ab225636.html;
56) Anti- human/mouse TAP, https://www.ptgcn.com/products/TAP1-Antibody-11114-1-AP.htm;
57) Anti-mouse PD-L1, https://www.cellsignal.cn/products/primary-antibodies/pd-l1-d5v3b-rabbit-mab/64988.

Eukaryotic cell lines

Policy information about cell lines and Sex and Gender in Research

Cell line source(s)	The EL4 mouse T cells were purchased from CTCC (China center for type culture collection, CTCC). The CAL27 human oral cancer, MDA-MB-231 human breast cancer, H1264 human lung cancer, MC38 mouse colon cancer, A375 human melanoma, and B16F10 mouse melanoma cells were purchased from ATCC.
Authentication	EL4, CAL27, MDA-MB-231, H1264, A375, and B16F10 were authenticated by the STR profiling analysis.
Mycoplasma contamination	All cells were without mycoplasma contamination.
Commonly misidentified lines (See ICLAC register)	No commonly misidentified cell lines were used.

Animals and other research organisms

Policy information about studies involving animals; ARRIVE guidelines recommended for reporting animal research, and Sex and Gender in Research

Laboratory animals	The stock# for all purchased animal strain were as follows: No.1103221911010423, No.1107301911000033, No.1107301911000034, No.1107301911000030, No.1107301911000057, and No.42010200003487. 8-week-old female C57BL/6 mice were housed in Specific Pathogen-Free (SPF) animal facilities (68-71.6°F temperature and 50%-60% humidity) for all animal experiments. The dark/light cycle animal rooms: 12 h of light and 12 h of dark. Experimental and control animals were housed in separate cages within the same room.
Wild animals	Wild animal is not used in this study.
Reporting on sex	The sex of the mice were female to minimize potential confounder, and the data analysis was single masked.
Field-collected samples	N/A.
Ethics oversight	The mice experiment was approved by the Ethics Committee for Animal Research, Stomatology of Wuhan University, China.

Note that full information on the approval of the study protocol must also be provided in the manuscript.

Plants

Seed stocks	N/A.
Novel plant genotypes	N/A.
Authentication	N/A.

Flow Cytometry

Plots

Confirm that:

☒ The axis labels state the marker and fluorochrome used (e.g. CD4-FITC).

☒ The axis scales are clearly visible. Include numbers along axes only for bottom left plot of group (a 'group' is an analysis of identical markers).

☒ All plots are contour plots with outliers or pseudocolor plots.

☒ A numerical value for number of cells or percentage (with statistics) is provided.

Methodology

Sample preparation	For analyzing the membrane level of PD-L1, MHC-I, and ICAM-1, tumor cells were collected and washed, followed by staining with anti-PD-L1, MHC-I, and ICAM-1 antibodies for 60 min on ice.<br>For analyzing tumor-infiltrating T cells from mice, the tumor samples were harvested for preparation of single cell suspensions. Red blood cells were lysed using ACK lysis buffer. Single cell suspensions were then incubated with anti-CD16/CD32 antibodies for 10 min, and then stained with a cocktail of antibodies for the surface markers for 30 min on ice. After that, cells were fixed and permeabilized, and subsequently stained for intracellular markers for 60 minutes on ice.
Instrument	CytoFLEX (Beckman Coulter, Life Sciences).
Software	FlowJo 10.4.2
Cell population abundance	N/A.
Gating strategy	Single cell gates based on FSC-H and FSC-A, and SSC-H and SSC-A were used to exclude non-singlets. A morphology gate based on FSC-A and SSC-A was used to exclude debris. For analyzing the levels of PD-L1 on cell surface, cells stained with isotype control antibodies were used to define the background non-specific staining, and then a PD-L1+ gate was used for cells stained with PD-L1.

☒ Tick this box to confirm that a figure exemplifying the gating strategy is provided in the Supplementary Information.
